# MLAA-34 knockdown shows enhanced antitumor activity via JAK2/STAT3 signaling pathway in acute monocytic leukemia

**DOI:** 10.7150/jca.46670

**Published:** 2020-09-30

**Authors:** Bo Lei, Lu Qian, Yanping Zhang, Yinxia Chen, Meili Gao, Walayat Shah, Xingmei Cao, Pengyu Zhang, Wanhong Zhao, Jie Liu, Jianli Wang, Xiaorong Ma, Yun Yang, Xin Meng, Fengmei Cai, Yan Xu, Jing Luo, Baiyan Wang, Yang Zhang, Aili He, Wanggang Zhang

**Affiliations:** 1 Second Affiliated Hospital, Medical School of Xi'an Jiaotong University, Department of Hematology, 157 Xiwu Road, Xi'an, Shaanxi, China.; 2 Department of Medical Research Center, Xi'an No.3 Hospital, the Affiliated Hospital of Northwest University, Xi'an, Shaanxi Province, China, 710008.; 3 Second Affiliated Hospital, Medical School of Xi'an Jiaotong University, Medical Laboratory, 157 Xiwu Road, Xi'an, Shaanxi, China.; 4 Department of Biological Science and Engineering, The Key Laboratory of Biomedical Information Engineering of Ministry of Education, School of Life Science and Technology, Xi'an Jiaotong University, Xi'an, Shaanxi Province, China, 710049.; 5 Institute of Basic Medical Sciences, Khyber Medical University, Peshawar, Khyber Pakhtunkhwa 25000, Pakistan.; 6 Xi'an No.4 Hospital, Department of Pathology, 21 Jiefang Road, Xi'an, Shaanxi, China.; 7 Second Affiliated Hospital, Shaanxi University of traditional Chinese medicine, Department of Hematology, 5 Wei Yang west road, Xianyang, Shaanxi, China.

**Keywords:** acute monocytic leukemia, MLAA-34, JAK2/STAT3, positive feedback loop

## Abstract

MLAA-34 is a novel leukemia-associated gene closely related to the carcinogenesis of acute monocytic leukemia (AML). MLAA-34 over expression has been observed to inhibit apoptosis *in vitro*. JAK2/STAT3 pathway plays an important role in cell proliferation, differentiation and inhibition of apoptosis in number of cancers. However, the relationship and interaction between MLAA-34 and JAK2/STAT3 has never been investigated in AML.

This study investigates and reports a novel relationship between MLAA-34 and JAK2/STAT3 pathway in AML both *in vitro* and *in vivo*. We constructed MLAA-34 knockdown vector and transfected U937 cells to observe its apoptotic activities in relation to JAK2/STAT3 signaling pathway *in vitro* and then *in vivo* in mouse model. Levels of expression of MLAA-34 and JAK2/STAT3 and its downstream targets were also measured in AML patients and a few volunteers.

We found that MLAA-34 knockdown increased U937 apoptosis *in vitro* and inhibited tumor growth *in vivo*. Components of the canonical JAK2/STAT3 pathway or its downstream targets, including c-myc, bcl-2, Bax, and caspase-3, were shown to be involved in the carcinogenesis of AML. We also found that the JAK2/STAT3 pathway positively regulated MLAA-34 expression. We additionally identified a STAT3 binding site in the MLAA-34 promoter where STAT3 binds directly and activates MLAA-34 expression. In addition, MLAA-34 was found to form a complex with JAK2 and was enhanced by JAK2 activation. Correlation of MLAA-34 and JAK2/STAT3 was further confirmed in AML patients.

In conclusion, MLAA-34 is a novel regulator for JAK2/STAT3 signaling, and in turn, is regulated by this interaction in a positive feedback loop. Thus we report a novel model of interaction mechanism between MLAA-34 and JAK2/STAT3 which can be utilized as a potential target for a novel therapeutic approach in AML.

## Introduction

Acute monocytic leukemia (AML) is a cancer of the myeloid lineage of blood cells. It is the most common acute leukemia, which results in white blood cells accumulation in the bone marrow with ultimate impaired hematopoiesis. Although the frequency of complete remission of AML has increased, its prognosis is usually poor due to its resistance to majority of the chemotherapeutic treatments 1-4. Therefore, it is important to seek novel agents or more effective therapies to overcome this cancer.

In the last two decades, immunotherapeutic approaches targeting leukemia-associated antigens (LAAs), have been indicated as a promising new strategy for AML patients 1, 5. We previously identified a novel gene monocytic leukemia-associated antigen-34 (MLAA-34, GenBank no. AY288977.2) in AML using serological analysis of recombinantly expressed cDNA clone (SEREX). We found that MLAA-34 exclusively reacted with sera from allogeneic leukemia patients but not with normal donor sera 6. Our previous study also demonstrated that the mRNA and protein levels of MLAA-34 were elevated in U937 cells and AML patients. Additionally, the expression levels of MLAA-34 have been found to be higher in M5 patients 5, 7.

MLAA-34 over expression markedly inhibited apoptosis in U937 cells 6. Bioinformatics findings showed that the regulation of the JAK-STAT cascade might be a novel interaction partner for MLAA-34 in U937 with MLAA-34 overexpressing cell line 7. STAT3 is a key signaling and transcription factor which is frequently activated by members of the Janus kinase (JAK) family 8-10. As a member of the JAK family, JAK2 generally transmits extracellular signals which further regulate a variety of cellular processes, such as immune responses, differentiation, hematopoiesis and growth 11. JAK2/STAT3 pathway is frequently upregulated in many human cancers. It plays a crucial role in cell proliferation, differentiation and anti-apoptosis 12,13. However, to the best of our knowledge, the identification of the possible interaction mechanism between MLAA-34 and JAK2/STAT3 in AML has never been investigated.

Thus, the objective of this study was to determine the interaction mechanism between MLAA-34 and JAK2/STAT3 pathway in AML. We functionally validated the role of MLAA-34 knockdown and its effects on JAK2/STAT3 pathway. JAK2 activator and inhibitor were used to assay the relationship between JAK2/STAT3 and MLAA-34. The interaction between MLAA-34 gene promoter and STAT3 was additionally certificated. Finally, this relationship between MLAA-34 and STAT3/JAK2 was validated in AML patients. Our findings provide new mechanistic insights into the crucial roles of MLAA-34 in regulation of JAK2/STAT3 pathway. Simultaneously, knockdown of MLAA-34 plays an important tumor-inhibiting function through JAK2/STAT3 signaling. These findings provide a novel intervention target for clinical implications in AML treatment.

## Materials and Methods

### Reagents and antibodies

Antibodies, including MLAA-34, STAT3, JAK1, JAK2, c-Myc, Bax, Caspase-3, bcl-2, IgG, β-actin, GAPDH, secondary mouse and rabbit antibodies, were purchased from Cell Signaling Technology, Shanghai, China. Annexin V-FITC/PI Bradford Reagent Kit was bought from Roche, USA. AG490 was purchased from Calbiochem-Nova- biochem, Bad Soden, Germany. IL-6 was purchased from Raybiotech, Inc., Norcross, GA, USA. Quick Change Site-directed Mutagenesis Kit (Stratagene, Shanghai, China), ChIP and EMSA Assay Kits (HyClone, Logan, Utah, USA) were used in the experiment. The pGCSIL-GFP, pcDNA3.1, pGL3-Basic/LUC, pRL-TK vectors, were purchased from Clontech Corporation. Reagents of cell culture were purchased and authenticated from Gene Chem Inc (Shanghai, China). Bradford Protein Assay Kit, Nu Page 4-12% Bis-Tris protein gels, ECL reagent used were Bio-Rad, Invitrogen, and Amersham Biosciences, respectively. Other reagents were bought from Sigma Aldrich, USA.

### Patients and sample selection

In this study, 41 AML patients (Supplemental Table 1) enrolled in the Department of Hematology of the Second Affiliated Hospital of Xi'an Jiaotong University from October 2011 to October 2016 were assayed for MLAA-34, JAK2, and STAT3 mRNA levels. An additional 6 samples from healthy volunteers (Supplemental Table 1) were collected as controls. All patients were treated according to the protocols and therapies as previously described 5. Peripheral blood samples (1 mL) were collected after informed consent was provided to further study according to the Ethics Committee of the School of Medicine, Xi'an Jiaotong University. All the study methodologies were conformed to the standards set by the Declaration of Helsinki.

### Cell culture

U937 cells (human leukemia cell line, ATCC) were cultured in RPMI-1640 medium, and HEK293 cells were cultured in DMEM. Additionally, U937 cells were treated with the JAK2 inhibitor AG490 (0-50 μM) for 6-24 h or the JAK2 activator IL-6 (0-40 ng/ml) for 12-48 h.

### RNA interference

MLAA-34 knockdown lentivirus was generated in the pGCSIL-GFP vector (KD), referred to as pGCSIL-GFP-MLAA-34. An empty pGCSIL-GFP vector was used as a normal control (NC). The siRNA sequence for MLAA-34 was 5′-GTACGTGGAGTTGTCAACA-3′. Following U937 cells were cultured in a 24-well plate for 24 h. The viral stock with a multiplicity of infection (MOI) of 100 was added and the transfection mixture was replaced with normal complete growth medium. The infected cells were referred to as MLAA-34-shRNA/U937 (KD) and Vec/U937 (NC) cells.

### Establishment of NOD/SCID mouse model

NOD/SCID mice (female, aged 6~8 weeks) were purchased from Vital River Laboratories (VRL, Beijing, China). The mice were housed and had free access to food and drinking water in the SPF laboratory. All animal care and euthanasia were performed in accordance with the guidelines of our Institutional Animal Care and Use Committee (IACUC), University Laboratory Animal Research. Previous KD and NC cells (3×10^7^ cells/200 μl) were injected into the caudal veins of female NOD/SCID mice (10/each group). We also subcutaneously injected mice with cyclophosphamide (100 mg/kg) for 5 days continuously before cell injection. Tumor size was assessed once per week, and tumor volume was calculated as previous description 14. Tumors were excised, and some of the tumors were fixed in formalin and paraformaldehyde to obtain frozen and paraffin-embedded sections, which were further subjected to immunohistochemical analysis. The rest of the tumors were subjected to RT-PCR and western blot analysis.

### Cell proliferation and apoptosis assay

The proliferation ability of infected U937 cells was detected by cell counting methods. The cell density was adjusted to 2×10^4^ cells/ml and inoculated in 96-well plates with 100 µl/well. The cells were counted after culture for 1, 2 and 3 days.

Apoptosis was analyzed after the infected U937 cells were cultured for 48 h. Fluorescein annexin V-FITC/PI double labeling was performed to detect the apoptotic effects in the infected U937 cells. The cells were stained with annexin V-FITC and PI. The apoptotic cells were quantified with a FACS Calibur flow cytometer (BD Biosciences) and analyzed with CELLQUEST software (BD Biosciences) 1.

### Western blotting

For the western blot assay, infected U937 cells were cultured for 48 h and were collected. The protein concentration was measured using a Bradford protein assay kit. Fifty micrograms of protein were loaded in NuPage 4-12% Bis-Tris protein gels and were detected by ECL reagent 5. Antibodies, including MLAA-34, STAT3, JAK1, JAK2, c-Myc, Bax, caspase-3, bcl-2, IgG, β-actin, GAPDH, secondary mouse and rabbit antibodies, were used in the experiment.

### Transcription factor regulation of MLAA34 gene expression

The primers for STAT3 were as follows: F: 5′-CCG*GAATTC*CGGAGAAACAGTTGGGACC-3′, R: 5′-CGG*GGTACC*CGTTCTCAGCTCCTCACATG-3′. Total RNA was extracted from U937 cells, and RT-PCR and PCR were used to amplify the STAT3 gene. The amplified STAT3 fragment and the pcDNA3.1 vector were digested with EcoRI and XhoI and then ligated and transformed into E. coli DH5α. After resistance selection and sequence, the recombinant plasmid was named as pcDNA3.1-STAT3, and an empty pcDNA3.1 vector was used as a control. Under the mediation of cationic liposomes, pcDNA3.1-STAT3 and the empty vector pcDNA3.1 were transfected into U937 cells for 24 h. Then, RT-PCR and western blotting were used to assay the expression of MLAA-34 and STAT3 in U937 cells.

To analyze the interference of the transcription regulator of STAT3 on MLAA-34 expression, the interference region was selected. STAT3 siRNA sequences were designed as follows: F: 5′-GCAGCAGCUGAACAACAUG-3′, R: 5′-CAUGUUGUUCAGCUGCUGC-3′. Similarly, under the mediation of cationic liposomes, si-STATT3 and NC-siRNA were transfected into U937 cells for 24 h. The expression of MLAA34 and STAT3 in the transfected cells was detected by RT-PCR and western blotting.

To further verify the STAT3 binding site on the MLAA34 gene core promoter, a mutant active detection vector with EGFP was constructed and named pGL3-Basic/EGFP/sSTAT3 (mut-STAT3). The core region of the MLAA-34 promoter with EGFP was constructed and named pGL3-Basic/EGFP/MLAA-34-wt (WT) and used as the positive control. The two constructed plasmids were transfected into U937 cells for 24 h, and the expression of green fluorescent protein was observed under a fluorescence microscope.

### Site-directed mutagenesis and luciferase assays

The core region in the MLAA-34 promoter was predicted and amplified by PCR using the following primers: F: 5′-GGGGTACCAAACCTCCCGAGCGCAGGTGCC-3′, R: 5′-CCAAGCTTAACAGCTGCGCCACCACTCCAGT-3′. The recombinant plasmid was named pGL3-Basic/LUC/MLAA-34-wt (wild type, core region in MLAA-34 promotor). According to the sequence (GGAAGG) of the STAT3 binding site in the core region of the MLAA-34 promoter, primers with site-specific mutations were generated using a QuickChange site-directed mutagenesis kit with the following sequences: F: 5′-TCTACCGGGGATGAGACTCTTCGTAACCTCCGCCGG-3′, R: 5′-CCGGCGGAGGTTACGAAGAGTCTCATCCCCGGTAGA-3′. The pGL3-Basic/LUC/MLAA-34-wt plasmid was used, and PCR was performed according to the manufacturer's protocol. Then, the PCR products of the STAT3 mutation were confirmed by sequencing. The site-specific mutagenized plasmid was named pGL3-Basic/LUC/mut-STAT3 (mut-STAT3).

HEK293 and U937 cells were inoculated in a 24-well culture. Then the pGL3-Basic/LUC/mut-STAT3 plasmid was cotransfected into NIH3T3 cells with 2 ng Renilla luciferase vector of pRL-TK as an internal interference. The same ratio of pGL3-Basic/LUC (negative control, NC group), pGL3-Promotor vector (positive control, PC group) and pGL3-Basic/LUC/MLAA-34-wt (wild type, WT group) vectors together with the Renilla luciferase vector of pRL-TK were also cotransfected into U937 and HEK293 cells for 24 h. The relative luciferase activity (RLA) was calculated by normalizing the fluorescent luciferase with the internal standard Renilla luciferase.

### Chromatin immunoprecipitation (ChIP) assay

ChIP was performed according to the manufacturer's instructions. Briefly, 1% formaldehyde was used to crosslink 1×10^7^ cells for 10 min and cells were then washed with ice-cold PBS, harvested by scraping and suspended in lysis buffer containing protease inhibitors. Following incubation on ice, 500 bp DNA fragments were formed under sonication. Anti-STAT3 was incubated with the supernatants with centrifugation for 10 min at 4 °C.

IgG or no antibody was used as a control. After using salmon sperm DNA/protein A-agarose to precipitate the immune complexes, they were recovered, and eluted with the ChIP elution buffer and reverse cross-linked by heating at 65 °C for 4 h. A small chromatin-protein sample was excluded and used as an input sample for the positive control in the PCR reaction. Extracted DNA was used as a template for PCR amplification. The primers for the STAT3 binding site in the MLAA-34 gene promoter or the control region were as follows: STAT3 sense: 5′-AGCCTTCATCTCAACCACAAC-3′ and antisense: 5′-CTCCCATGTAGTGATCGGTTT-3′; control sense: 5′-AGGCTGGTCTTGAACTCCTGA-3′and antisense: 5′-GTGTGAACGCTGGTGAGAGC-3′; both at 60 °C for 30 cycles 15.

### Electrophoretic mobility shift assay (EMSA)

Nuclear extracts were prepared and incubated with biotin-labeled wt or mutant probes. DNA-protein complexes were separated and transferred to nylon membranes, and shifted probes were detected with HRP-conjugated streptavidin. The probe was 5′-AGCGCAGGTGCCCTCTGGCCGGGAAGTACTTCACCAT-′3. After incubation for 20 min at room temperature, 4% native polyacrylamide gel was used to separate the samples. For competition analysis, STAT3 proteins were incubated with unlabeled probes for 20 min at room temperature before the addition of labeled probes 16.

### Microarray analysis and quantitative real-time PCR (qRT-PCR)

Total RNA from NC and KD cells was extracted using TRIzol reagent. Microarray analysis was performed using an Affymetrix microarray human genome U133 chip containing 40,000 genes. Microarray data were further analyzed by ingenuity pathway analysis (IPA). Isolated RNA was assay for the mRNA levels of STAT3, JAK2, c-myc, Bax, caspase-3 and bcl-2 using RT-PCR kits, as in our previous study 1. The primer sequences were as follows: STAT3 sense: 5′-CTGAA CTTCGGGGTGATCGG-3′ and antisense: 5′-GGCTTGTCACTCGAATTTGAGA-3′; JAK2 sense: 5′-CTGAGTTGACTCCTACTGTGGA-3′and antisense: 5′-TCTTCCCAGGGTCGATAAAGT-3′; c-Myc sense: 5′-GATTCTCTGCTCTCCTCGAC-3′ and antisense: 5′-TCCAGACTCTGACCTTTTGC-3′; Bax sense: 5′-GCATCGCTTCGGGGTGAT-3′ and antisense: 5′-CGTAGCGAAGCCCACTA-3′; bcl-2 sense: 5′-AGAAGGGTCCCAGCTA-3′ and antisense: 5′-TTCGGGGTGATCGG-3′; and caspase-3 sense: 5′-CTCGGTCTGGTACAGATGTCGATG-3′ and antisense: 5′-GGTTAACCCGGGTAAGAATGTGCA-3′. The internal reference β-actin primer sequences were as follows: sense: 5′-AGAGGGAAATCGTGCGTGAC-3′, antisense: 5′-CAATAGTGATGACCTGGCCGT-3′.

### Coimmunoprecipitation

Co-immunoprecipitation (CO-IP) was used to analyze the interaction between MLAA-34 and JAK2. Briefly, U937-MLAA-34 cells were cultured and transfected with pGCFU-MLAA-34 vectors to generate U937-MLAA-34 cells. Lysis buffer was added to the transfected cells. A protein agarose A+G of 30 μl and 0.5 µg of other antibodies of the same species were added to react for 2 h at 4 °C and centrifuged at 3000 rpm for 15 min. The antibodies were added to the supernatant and incubated for 12 h. A protein agarose A+G of 30 μl was added and incubated for 3-6 h and centrifuged at 3000 rpm for 5 min. The supernatant was discarded, and 1ml lysate was added to react for 10 min. The precipitate was collected, and 30 µl 2× sample buffer was added and placed in boiling water for 5 min. The tubes were immediately placed on ice to cool and then centrifuged at 12000 rpm for 10 min. A 30 µl sample was used for western blotting.

### Immunohistochemistry

Immunohistochemistry (IHC) was performed on 4 μm sections from frozen tumor xenografts of mice as described previously using bcl-2 (1:100), Bax (1:100), JAK2 (1:100), and STAT3(1:100) antibodies for 60 min at room temperature or overnight at 4 °C. Slides were developed as described previously 4. Then, the slides were mounted and observed in three random microscopic fields/tumors.

### Statistical analysis

All data were expressed as the mean ± S.D. The Mann-Whitney U test was used to evaluate the differences between groups. Spearman's correlation analysis was used for AML patient data. *P-*values were generated using unpaired Student's *t*-test and considered significant at *P* ≤ 0.05, and all experiments were performed in triplicates.

## Results

### MLAA-34 knockdown inhibits tumor growth *in vitro* and *in vivo*

**Figure 1:**
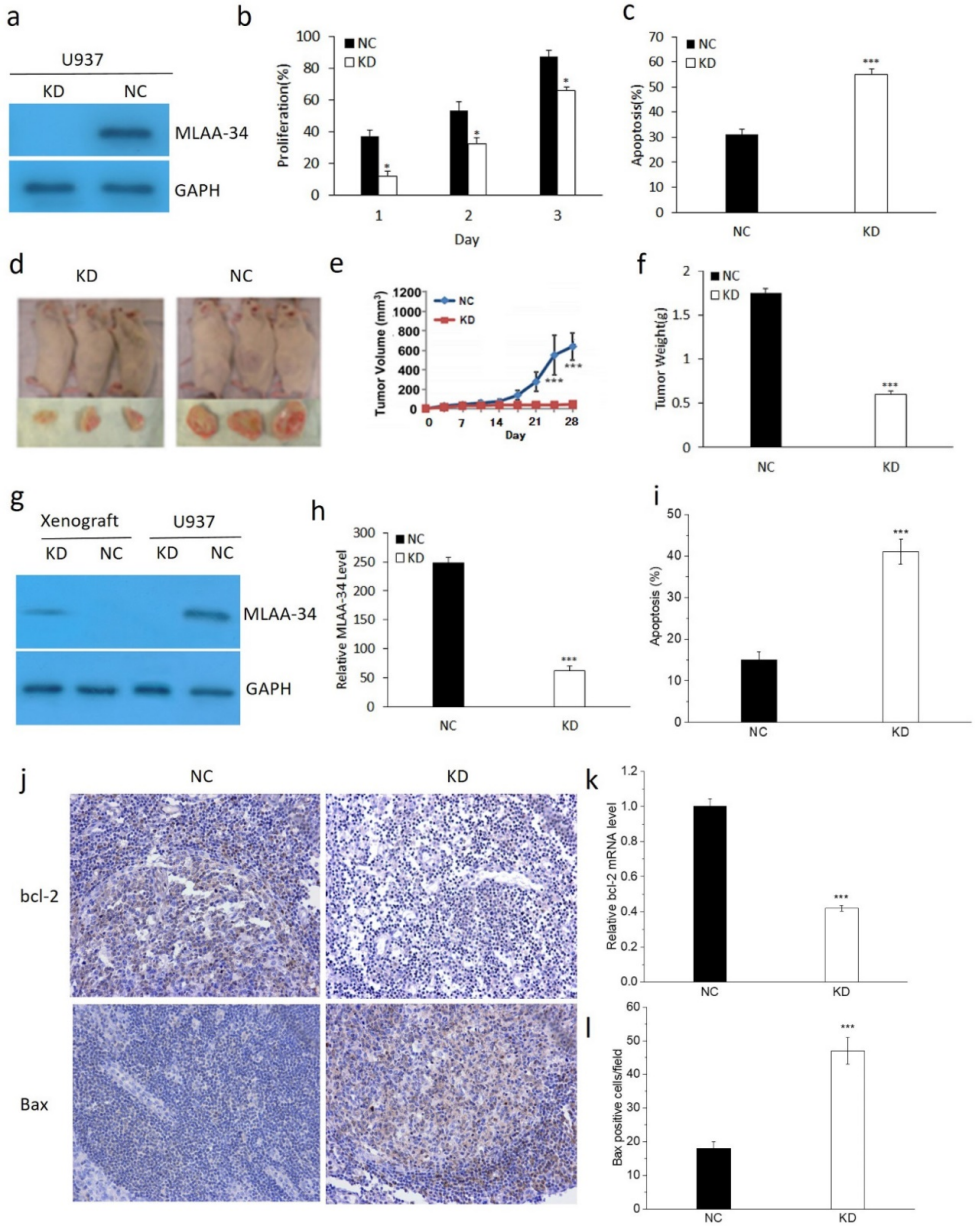
** Effect of MLAA-34 knockdown *in vitro* and *in vivo*.** For *in vitro* experiment, **(a)** MLAA-34 expression was analyzed by western blotting with an anti-MLAA-34 antibody. GAPDH was used as a loading control. **(b)** Proliferation of the cells was measured at 1, 2, and 3 days as a percentage increase with respect to the control (day 0). **(c)** The cells were stained with annexin V-FITC and PI according to the manufacturer's instructions. For *in vivo* experiment, U937/KD and U937/NC cells were injected into the caudal veins of female NOD-SCID mice. Mice were also injected subcutaneously with 100 mg/kg of CTX for 5 days continuously before cell injection. Tumors were measured by digital calipers weekly for 4 weeks, and tumor volume was determined using the following formula: length ×(width)^2^/2. After 28 days, the tumors were harvested, fixed, and spleen tissues were evaluated for apoptosis. **(d)** Representative images of NOD-SCID mice bearing tumors. **(e)** Tumor volume. **(f)** Tumor weight upon harvesting at day 28. (**g**) MLAA-34 western blotting in U937 cells and NOD/SCID mice. **(h)** Real-time PCR analysis of MLAA-34 expression in the engrafted tumors. **(i)** Apoptotic percentage. **(j)** Immunohistochemical results of the levels of bcl-2 and Bax in spleen tissues. **(k)** Real-time PCR analysis of bcl-2 gene expression in the xenografted tumors.** (l)** The average number of positive cells/field after counting Bax-positive stained cells from three random fields on the slide. All experiments were repeated in triplicate. The results are expressed as the mean ± SD;^*^P<0.05 and ^***^P<0.001 vs NC group.

To determine the role of MLAA-34, the U937 cells were transfected with the KD and NC vectors. As shown in **Figure 1**, MLAA-34 was successfully knocked down compared with the vector control (**Figure 1A**). We found that MLAA-34 knockdown significantly decreased (*P*<0.05) cell proliferation in a time-dependent manner (**Figure 1B**). In addition, apoptosis was significantly increased (*P*<0.001) in KD cells compared with NC cells (**Figure 1C**). These results demonstrated that knockdown of MLAA-34 played an important role in inhibiting the proliferation of U937 cells.

To evaluate the tumor-promoting or proliferation effects of MLAA-34 *in vivo*, we determined the tumorigenic potential of U937 cells in a NOD/SCID mouse model. We observed that tumors in mice that received KD cells grew much less than tumors that received NC cells (**Figure 1D**). Tumor volumes were significantly decreased (*P*<0.001) in KD mice compared with those in NC mice after 21 days (**Figure 1E**). Correspondingly, tumor weight was significantly decreased (*P*<0.001) in KD mice (**Figure 1F**). Similarly, the results of MLAA-34 protein expression levels indicated that MLAA-34 was knocked down (**Figure 1G**) and indicated a significant reduction (*P*<0.001) in mRNA expression levels in the KD group (**Figure 1H**). Apoptotic cells were significantly increased (*P*<0.001) in KD mice compared with NC mice (**Figure 1I**). Additionally, obvious reduction in bcl-2 (**Figure 1J & K**) and increment in Bax (**Figure 1J & L**) were observed in splenic tissues of KD mice compared to NC mice. These results extend the *in vitro* findings of tumor-suppressive capabilities of MLAA-34 knockdown.

### MLAA-34 knockdown mediates associated genes involved in the JAK2/STAT3 pathway

**Figure 2:**
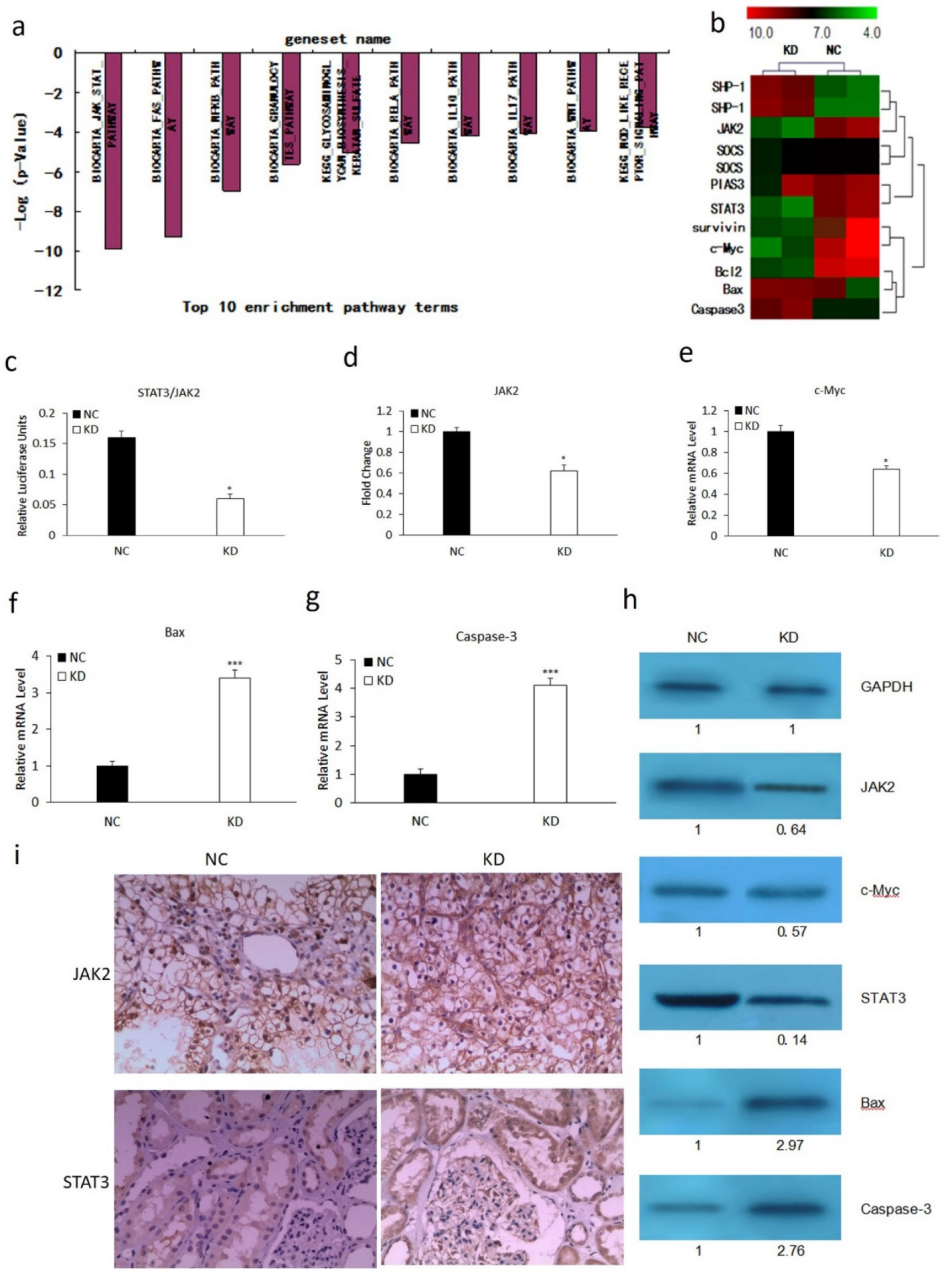
** Effect of MLAA-34 knockdown on the expression of genes involved in the JAK2/STAT3 pathway. (a and b)** Total RNA from NC and KD groups was analyzed by Affymetrix Human Genome U133 chip. Gene ontology studies using IPA analysis of microarray data. **(c-g)** RT-PCR for STAT3, JAK2, c-Myc, Bax and Caspase-3. **(h)** western blotting of JAK2, STAT3, c-Myc, Bax, and caspase-3 antibodies. GAPDH was used as a loading control. The values below the blots show relative intensity obtained after densitometric analyses.** (i)** Kidney and** (j)** spleen tissues derived from KD or NC mice by immunohistochemical staining for JAK2 and STAT3. All experiments were repeated three times, and a representative experiment is shown. The results are expressed as the mean ± SD, *P< 0.05, ***P< 0.001 vs NC group.

**Figure 3:**
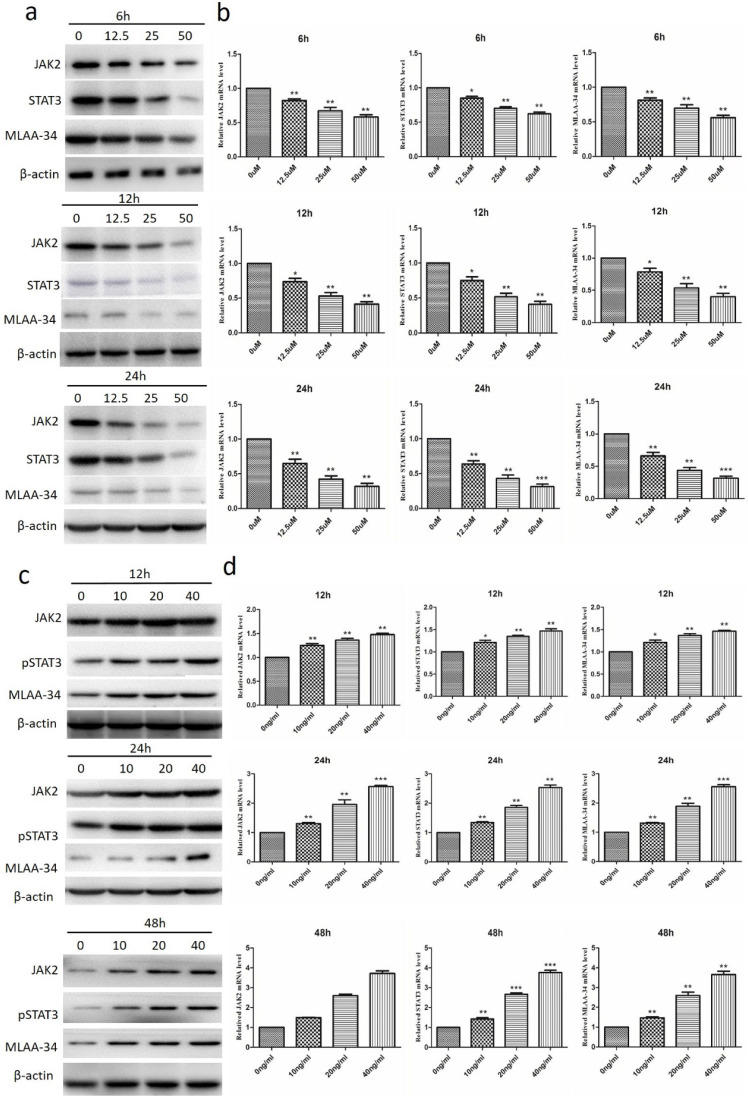
** Effect of the inhibitor of JAK2 (AG490) and the activator of JAK2 (IL-6) on the expression of MLAA-34 and the JAK2/STAT3 pathway in U937 cells. (a-b)** AG490 treatment at concentrations of 0, 12.5, 25, and 50 µM for 6, 12, and 24 h. **(c-d)** IL-6 treatment at concentrations of 10, 20, and 40 ng/ml for 12, 24, and 48 h. Western blotting (results of a, c) and qRT-PCR (results of b, d) were used to assay the protein and mRNA expression levels. Data represent the mean ± SD from at least three independent experiments. *P<0.05, **P< 0.01, and ***P< 0.001 vs the corresponding control.

We performed microarray analysis using an Affymetrix gene chip to explore the mechanism involved in MLAA-34-knockdown-mediated tumor suppression. We found that MLAA-34 knockdown significantly affected cellular pathways involved in carcinogenesis. The relative enrichment pathway terms were JAK-STAT, Fas, NFKB and so on (**Figure 2A**). This signified the importance of MLAA-34 in AML progression. Additionally, MLAA-34 knockdown mediated genes that are either components of the JAK2/STAT3 pathway or its downstream targets (**Figure 2B**). Important components of this pathway that were differentially regulated in KD cells compared to those in NC cells, included STAT3 (~7-fold downregulated) and JAK2 (~2.5-fold downregulated). The downstream targets of the JAK2/STAT3 pathway included proto-oncogenes, such as c-Myc (2-fold downregulation), survivin (~3.5-fold downregulation) and antiapoptotic genes of bcl-2 (~3.5-fold downregulation). Apoptotic genes of Bax and caspase-3 were upregulated (2- to 4-fold upregulation). Next, we investigated the variations of JAK2/STAT3 and these involved genes by qRT-PCR and western blotting. The results showed a significant reduction in the expression levels of JAK2, STAT3 and c-Myc in KD cells (**Figure 3C-E & H**). We observed a significant increment in the expression levels of Bax and caspase-3 (**Figure 2F-H**). JAK2, STAT3 were also confirmed by IHC analysis in tumors derived from kidney (**Figure 2I**) tissues. As shown in Figure 2h-i, expression levels of JAK2 and STAT3 reduced in the KD group, especially in kidney tissues. These results confirm that MLAA-34 knockdown in acute monocytic leukemia cells downregulates the JAK2/STAT3 pathway and its downstream genes.

### Positive correlation between JAK2/STAT3 pathway and MLAA-34 in U937 cells

To investigate the correlation between the JAK2/STAT3 pathway and MLAA-34, we measured the effect of JAK2 inhibitor and activator on the expression of MLAA-34 and JAK2/STAT3. As shown in **Figure 3A**, treatment of U937 cells with the JAK2 inhibitor AG490 at concentrations of 12.5, 25, and 50 μM for 6, 12 or 24 h significantly reduced both MLAA-34 protein and mRNA expression levels. The expression levels of JAK2 and STAT3 were also significantly decreased (**Figure 3A & B**). When we treated U937 cells with the JAK2 activator IL-6 (10, 20 and 40 ng/ml) for 12, 24 or 48 h, marked increments in MLAA-34, JAK2 and pSTAT3 expressions were observed (**Figure 3C & D**). The findings demonstrated that the JAK2/STAT3 pathway is positively correlated with MLAA-34 expression. This positive regulation effect was observed in a dose-dependent manner in this experiment.

### STAT3 positively regulates MLAA-34 expression by binding directly to the MLAA-34 promoter

**Figure 4:**
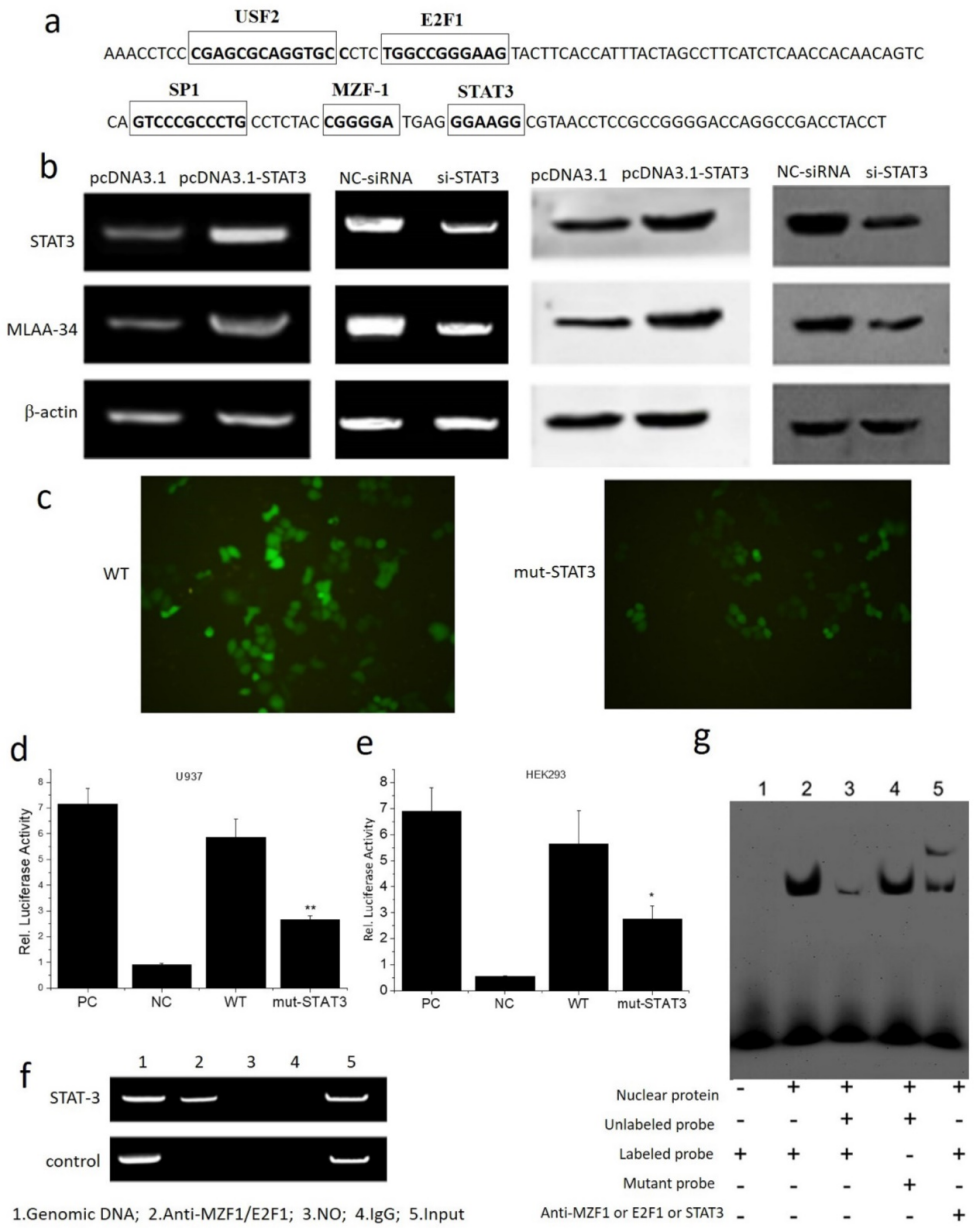
** Interaction between MLAA-34 and the STAT3 binding site in the MLAA-34 gene. (a)** Sequence of the proximal MLAA-34 gene promoter. Transcription factors, such as USF2, E2F1 and SP1, which have been predicted for the MLAA-34 gene by computer-assisted analysis, are boxed. The new putative binding site for STAT3 is also boxed. **(b)** STAT3 overexpression and siSTAT3 on MLAA-34 expression levels. **(c)** STAT3 mutant active detection vector with EGFP on the expression of green fluorescent protein.** (d-e)** Regulation of the STAT3 binding site in the MLAA-34 promoter by mut-STAT3 in U937 and HEK293 cells. **(f)** Binding of STAT3 to the STAT3 binding site in the MLAA-34 gene promoter *in vitro* determined by ChIP assay. Genomic DNA and input chromatin (input), which represent portions of sonicated chromatin before immunoprecipitation, were both used as positive controls.** (g)** STAT3 binding site in the MLAA-34 gene promoter *in vitro* was measured by EMSA. *P< 0.05 and **P< 0.001 for all experiments as calculated from Student's t test.

The interaction between MLAA-34 and STAT3 was assayed. Based on the transcription factor (TF) prediction programs Gene-regulation, Transfac6.0 and ALGGEN-PROMO, several putative TF binding sites were identified, including USF2, E2F1, SP1, MZF-1 and STAT3 at the MLAA-34 promoter. The binding site for the STAT3 transcription factor is 5′-GGAAGG-3′ (**Figure 4A**). Overexpression of STAT3 enhanced MLAA-34 expression in U937 cells, while siRNA-mediated knockdown of STAT3 reduced MLAA-34 expression (**Figure 4B**). When the binding site of STAT3 in the MLAA-34 gene promoter was mutated, the expression of GFP decreased (**Figure 4C**). The role of the STAT3 binding site within the MLAA-34 gene promoter was further demonstrated using a luciferase reporter assay. As shown in **Figure 4D & E**, we found that in both U937 and HEK293 cells, the mutation of the STAT3 site (mut-STAT3) resulted in reduction of luciferase activity (*P*<0.05 and *P*<0.01). Furthermore, ChIP and EMSA were performed to verify whether STAT3 was directly bound to the STAT3 binding site in the MLAA-34 gene promoter. U937 cell lysate was immunoprecipitated with anti-STAT3 antibody, and the fragment containing the STAT3 binding site in the MLAA-34 gene promoter was amplified by PCR (**Figure 4F**). As shown in Figure 4g, EMSA was used to confirm the interaction between STAT3 and MLAA-34. The labeled probe designed for the STAT3 binding site in the MLAA-34 gene promoter was incubated with nuclear protein, unlabeled probe or mutated probe, and band shifts were detected, but not the target band shift. However, in labeled probe incubation with nuclear protein and anti-STAT3, the second and target band shifts were detected. The results of ChIP and EMSA assays indicated that STAT3 binds directly to the MLAA-34 gene promoter. These results show that STAT3 is a positive transcription factor that regulates MLAA-34 expression.

### MLAA-34 interacts with endogenous JAK2, and JAK2 activation enhances this interaction

**Figure 5:**
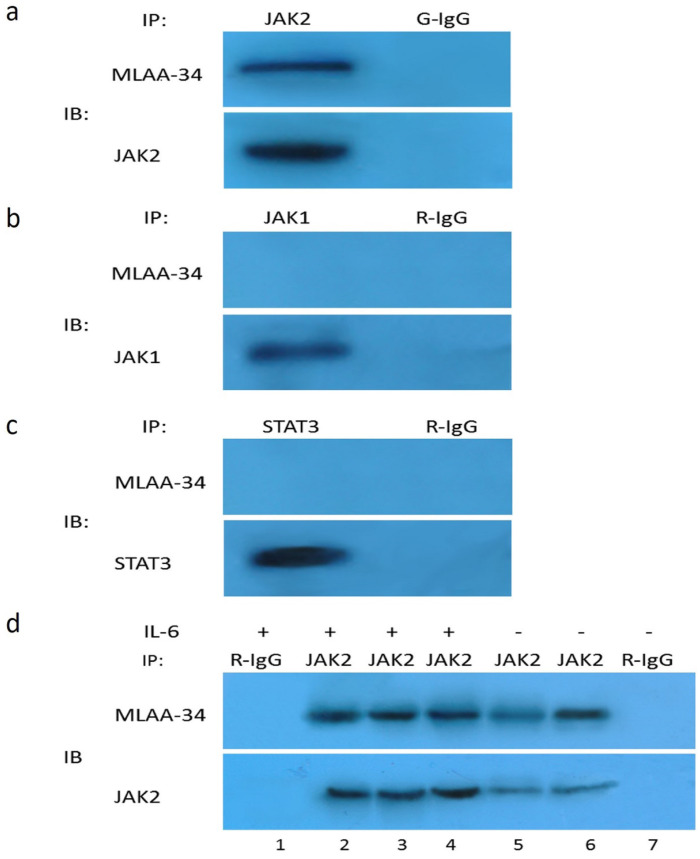
** Co-IP of endogenous JAK2 with MLAA-34 *in vitro*.** Lysates of U937-MLAA-34 cells were immunoprecipitated with anti-JAK2 and normal goat IgG (G-IgG, negative control) **(a)** or with anti-STAT-3, anti-JAK1, or normal rabbit IgG (R-IgG, negative control) **(b, c). (d)** U937-MLAA-34 cells were untreated or treated with 5 ng/mL of IL-6 for 15 min. Cell lysates were immunoprecipitated with the indicated antibodies.

Our results thus far suggest that JAK2 activation may be directly or indirectly dependent on the presence of MLAA-34. Therefore, we tested the possible interaction between endogenous JAK2 and MLAA-34 using the co-IP method. Based on theU937-MLAA-34 cells, we found that MLAA-34 formed a complex with endogenous JAK2 (**Figure 5A**) but not JAK1 or STAT3 (**Figure 5B & C**). This interaction was enhanced following treatment with the JAK2 activator IL-6 (**Figure 5D**). Our results indicate that MLAA-34 interacts with JAK2 and that MLAA-34 is required for cytokine-induced JAK2 activation.

### Variable expression of MLAA-34 and positive correlation with JAK2/STAT3 in AML patients

**Figure 6:**
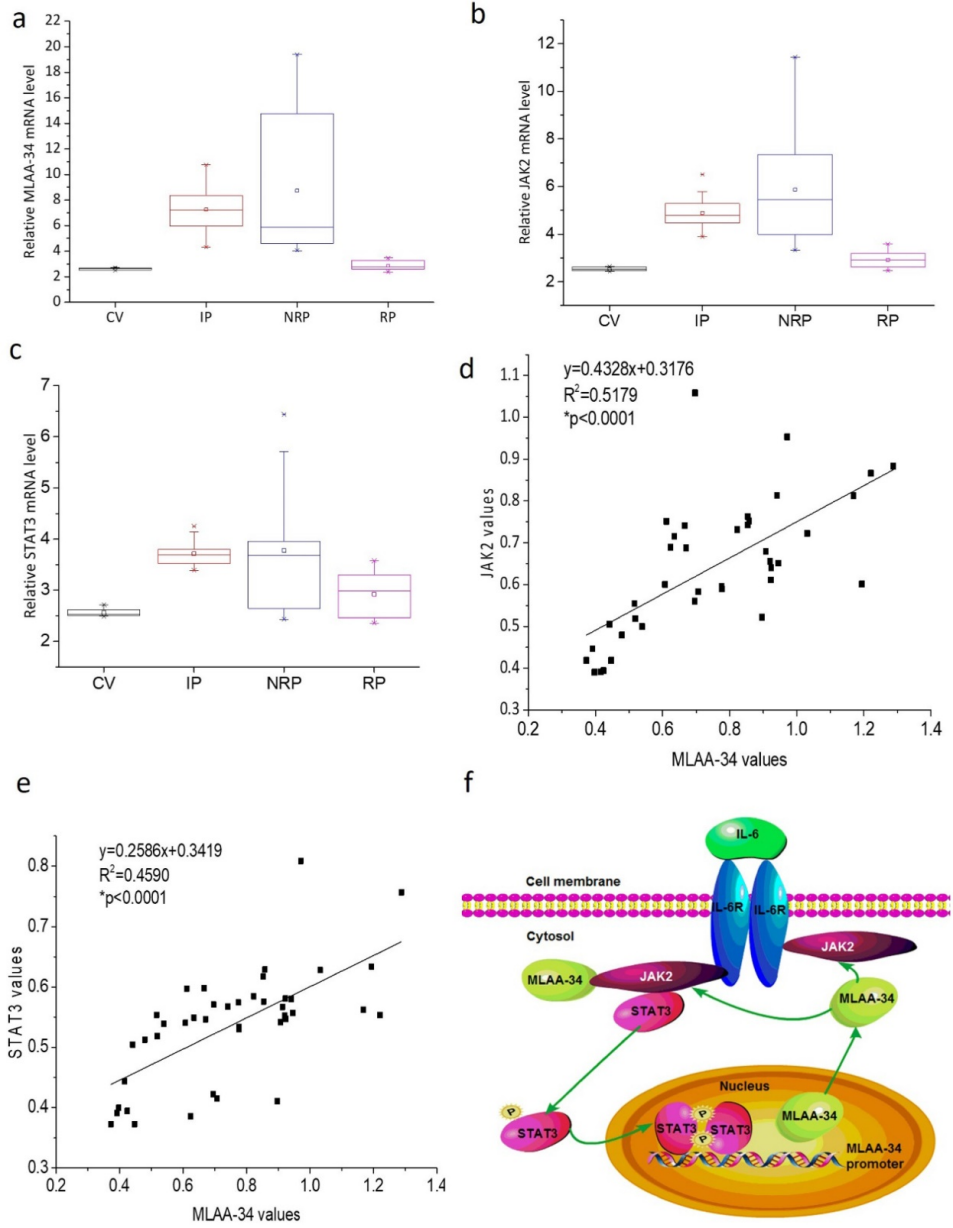
** MLAA-34 expression is variable and is positively correlated with JAK2/STAT3 in AML patients. (a-c)** Box plot representing mRNA expression levels of MLAA-34, JAK2, and STAT3 in AML patients and volunteers (n=47). Expression levels were calculated using the 2^-∆Ct^ method relative to β-actin expression. Spearman's correlation analysis of MLAA-34 mRNA levels and JAK2 mRNA levels **(d)** and STAT3 **(e)** in AML patients (n=41), calculated by the 2^-∆Ct^ method relative to β-actin expression and expressed as log10. **(f)** Schematic diagram of a reciprocal positive feedback regulation and the interaction between MLAA-34 and JAK2/STAT3 signaling in AML carcinogenesis.

To further verify the correlation of MLAA-34 and JAK2/STAT3, we analyzed the mRNA levels of MLAA-34, JAK2, and STAT3 in AML patients and healthy volunteers. In this study, 41 AML patients, including 17 incipient patients (IP), 14 nonremission patients (NRP), and 10 remission patients (RP), and 6 healthy control volunteers (CV) were analyzed. The clinical characteristics at diagnosis were shown in Supplemental Table 1. As shown in **Figure 6A-C**, similar changes in the mRNA expression levels of the examined genes were observed in different AML patients. Relatively higher expression levels were observed in the NRP group than in the IP group of patients, followed by the RP group compared to those in the CV group. Correlation analysis indicated that MLAA-34 mRNA expression is positively correlated with JAK2 and STAT3 in AML patients (**Figure 6D & E**).

Based on previous findings, we propose an interaction and a schematic diagram of a positive feedback regulation between MLAA-34 and JAK2/STAT3 signaling in AML carcinogenesis, as shown in **Figure 6F.**

## Discussion

MLAA-34 is a novel M5-associated antigen that is highly expressed in U937 cells and M5 patients. However, little is known about the key regulatory proteins and signaling pathways for MLAA-34. In this study, we revealed a positive feedback regulation between MLAA-34 and JAK2/STAT3. On the one hand, we observed that MLAA-34 knockdown decreases proliferation, increases apoptosis and inhibits tumor growth *in vitro* and *in vivo*. Down regulation of MLAA-34 decreases JAK2/STAT3 signaling as well as its downstream target genes. On the other hand, we found that JAK2/STAT3 signaling positively regulated MLAA-34. We found the STAT3 binding site in the MLAA-34 promoter. A complex formed between MLAA-34 and JAK2 was demonstrated, and this was enhanced by IL-6-mediated JAK2 activation (**Figure 6F**). To our knowledge, this is the first study that explores the interaction between MLAA-34 and JAK2/STAT3 signaling pathway and demonstrates its key role in AML.

Our previous studies revealed that MLAA-34 has a potential antiapoptosis effect and may be a novel antiapoptotic factor related closely to carcinogenesis or the progression of AML 1,2,4,7. Data from present investigation displayed that MLAA-34 knockdown significantly decreased proliferation, increased apoptosis, and prominently enhanced tumor inhibition. This additionally suggested an important function of MLAA-34 for apoptosis and proliferation in U937 cells and AML patients as in our previous studies 1,2,4,7. Reports have indicated that many types of cancer cells are prone to undergo apoptosis 17. Therefore, our findings suggest that down regulation of MLAA-34 may enhance U937 cell apoptosis.

JAK2/STAT3 signaling can induce cell proliferation, differentiation and antiapoptosis. Aberrantly activated JAK2/STAT3 signaling is critical in malignant progression by promoting cell growth. Inhibited growth and induction of apoptosis through inactivation of JAK2/STAT3 signaling in many cancer cells and tumors have been demonstrated in many published studies. 12,13,18. Therefore, the JAK2/STAT3 pathway is considered a target for anticancer therapy in many human cancers 10,18-20. Our findings revealed that knockdown of MLAA-34 were accompanied by the decreased expression of JAK2/STAT3 signaling molecules. This is also in agreement with the induction of apoptosis and antitumor effects by the down regulation of MLAA-34 in our previous studies 1,2,4,7. Our findings strongly suggest the fact of the positive correlation between MLAA-34 and JAK2/STAT3 pathway.

Cellular pathways affected by MLAA-34 knockdown are mainly correlated with cell apoptosis or proliferation processes, immune regulation and inflammation reaction, and KEGG pathways. This finding is similar to our previous findings of shotgun analysis of protein interactions with MLAA-34 7. In the present study, microarray data revealed that the JAK2/STAT3 pathway or its downstream targets, which included proto-oncogenes, antiapoptotic genes and apoptotic genes, were affected by MLAA-34 knockdown. In particular, c-Myc (2-fold downregulated), survivin (~3.5-fold downregulated), bcl-2 (~3.5-fold downregulated), Bax and caspase-3 (2- to 4-fold upregulated) were additionally found and validated. Down regulation of c-myc has often been observed in antitumor drugs or medicinal herb components, such as icaritin treatment in three leukemia cell lines and one primary AML bone marrow sample 2, 21. The over expression of antiapoptotic bcl-2 proteins is correlated with an overall lower survival rate for AML patients. In contrast, inhibition of bcl-2 has been reported to directly induce apoptosis in cultured AML cell lines and primary patient samples, forming an active bcl-2/Bax complex 22,23. Survivin is a member of the apoptosis inhibitor family. Suppressing the expression of survivin could inhibit cell proliferation in a tumor and improve the sensitivity to chemotherapeutic drugs, eventually inducing the apoptosis of tumor cells. Reports have demonstrated that silencing survivin could significantly reduce the proliferation of leukemia cells and induce apoptosis in FLT3 mutant mice 24-26. The survivin protein is able to inhibit caspases, which are essential in cells for apoptosis, or programmed cell death. Among caspases, caspase-3 is an important enzyme in apoptosis and the execution of apoptosis 26,27. Thus, our findings demonstrate that MLAA-34 knockdown is not solely responsible for the observed JAK2/STAT3 signaling but may cooperate with its downstream target genes.

The JAK2/STAT3 signaling pathway can be activated by several cytokines, including IL-6. IL-6-mediated activation of JAK/STAT3 signaling has been shown to increase the proliferation of cancer cells 28,29. It has been demonstrated that autocrine IL-6 production can induce the constitutive activation of STAT3, which plays a major role in STAT proteins in oncogenesis 30. However, AG490 can effectively block the activation of the JAK2/STAT3 signaling pathway in different cancer cell lines 28,31. In this study, to determine the correlation between MLAA-34 and JAK2/STAT3 pathway, an inhibitor of JAK2 (AG490) or an activator of JAK2 (IL-6) were treated in U937 cells. We found thatthe expression levels of the JAK2/STAT3 signaling pathway were also significantly inhibited or activated. Similarly, it has been reported that the abnormal expression of IL-6, IL-10 and TNF-α may lead to the activation of the JAK/STAT pathway 31. Previous studies have also demonstrated the inhibition by AG490 on the growth of malignant cells from patients with acute lymphocytic leukemia (ALL) and mycosis fungoides (MF) 30. Importantly, this inhibition or activation of JAK/STAT3 signaling was correlated with an evident reduction or increment of MLAA-34 expression. This provided the further evidence of positive regulation of JAK2/STAT3 signaling on MLAA-34 expression and a feedback loop that is formed between JAK2/STAT3 signaling and MLAA-34 (**Figure 6F**).

STAT3 belongs to a family of DNA-binding proteins that not only fulfill their roles in normal cell signaling but may also contribute to oncogenesis 32,33. It has been shown that gene transcription is regulated by STAT3 through direct interactions with the promoter regions of target genes 34. In the present paper, we firstly identified one candidate sequence for the STAT3 binding site in the MLAA-34 gene promoter, which indicated increase in transcriptional activity upon STAT3 regulation. Generally, STAT3 is activated by tyrosine phosphorylation, which induces its dimerization. This dimer binds to DNA target sites with about 9 base-pair bp (7-12 bp) consensus sequence of TTCCGGGAA (TTA/C^2-6^ NAA) 35-37. Thus, this binding site for STAT3 on the MLAA-34 promoter is noncanonical based on sequence comparisons. Using luciferase, ChIP and EMSA assays, we additionally demonstrated that this STAT3 binding site is both necessary and sufficient for transcription activity via the MLAA-34 promoter. Furthermore, using a Co-IP assay, we found that JAK2 interacted with MLAA-34. Moreover, JAK2 activation by IL-6 may enhance this interaction via the simultaneous increase in MLAA-34 and JAK2 proteins expression. These findings indicated that STAT3/JAK2 contributes to the activation of MLAA-34 expression. In our previous papers, we found that MLAA-34 was increased in U937 cells and M5 patients 5,7. In the present paper, we further certificated that the activation of STAT3/JAK2 produces an increase in the transcription level of MLAA-34in U937 cells and M5 patients. These findings warrant the support of the use of MLAA-34 in clinic trials. Based on these findings, we propose a novel model for the molecular events between MLAA-34 and JAK2/STAT3. In this molecular model, p-STAT3 binds to MLAA-34 gene promoter and stimulates the interaction of MLAA-34-JAK2. Conversely, this interaction further stimulates MLAA-34 and JAK2/STAT3 signaling (**Figure 6F**).

In conclusion, we revealed that MLAA-34 knockdown is accompanied by a decrease in the JAK2/STAT3 pathway. Simultaneously, JAK2/STAT3 enhances MLAA-34 activation. A positive feedback loop was formed between MLAA-34 and JAK2/STAT3 pathways. MLAA-34/JAK2/STAT3 signals modulate its downstream target genes in AML carcinogenesis. Our results provide the first evidence that MLAA-34 is central to JAK2/STAT3 signaling. We validated that the knockdown of MLAA-34 is highly like to potentiate an antitumor response, thus highlighting the JAK2/STAT3 pathway via MLAA-34 as a potential target for new therapeutic approaches in AML.

## Supplementary Material

Supplementary table S1.
