# Supplementary material for: MLAA-34 knockdown shows enhanced antitumor activity via JAK2/STAT3 signaling pathway in acute monocytic leukemia
Source: J Cancer. 2020 Sep 30;11(23):6768–81. doi: 10.7150/jca.46670 (PMC7592008; doi:10.7150/jca.46670)
Supplement: Supplementary file 1 — Supplementary table S1. [file jcav11p6768s1.pdf]

Supplemental Table 1 The clinical characteristics at diagnosis

Number	Sex	Age	WBC	Hb	PLT	M+P (%)
Incipient patient						
1	Female	62	29.01	79	9	43.5
2	Female	24	45.07	76	14	37.5
3	Female	36	1.38	56	15	90.0
4	Female	29	11.7	110	63	88.1
5	Male	52	77.93	116	101	61.0
6	Female	35	2.46	84	25	33
7	Male	32	1.06	71	114	67.5
8	Female	76	2.09	87	54	39.0
9	Male	60	36.65	103	13	75.0
10	Male	28	53.79	125	80	70.5
11	Male	56	31.19	56	16	86.0
12	Male	15	6.4	43	26	44.0
13	Male	19	59.80	56	71	73.5
14	Male	35	14.2	86	79	39.5
15	Male	58	34.64	70	11	34.5
16	Female	35	7.21	71	60	44.5
17	Male	45	4.93	88	68	52.5
Non remission patients of M5						
1	Male	62	32.7	86	291	23.5
2	Male	24	19.3	90	26	27.5
3	Female	36	11.19	32	43	40.0
4	Female	29	2.46	84	25	34.0
5	Male	52	1.06	114	71	31.0
6	Female	35	5.9	57	21	23.0
7	Female	48	3.55	87	256	27.5
8	Female	62	29.01	76	14	29.0
9	Male	46	1.05	83	3	25.0
10	Female	59	4.05	70	35	21.5
11	Male	56	14.5	73	46	26.0
12	Male	35	5.9	93	49	21.0

13	Male	19	9.5	96	71	33.5
14	Male	35	6.8	83	65	20.0
Remission patients of M5						
1	Male	31	2.53	121	107	1.5
2	Male	24	2.49	140	145	1.0
3	Female	48	4.24	94	245	2.0
4	Female	29	11.7	110	167	1.8
5	Male	46	4.07	128	256	1.0
6	Male	57	5.21	131	106	3.0
7	Male	32	4.67	117	114	1.5
8	Female	24	7.68	121	190	1.0
9	Female	45	11.47	96	254	2.0
10	Male	56	2.9	125	105	0.5
Control volunteers						
1	Male	47	8.44	215	151	1.0
2	Male	22	5.96	182	157	1.5
3	Male	33	6.14	133	149	2.0
4	Male	24	7.54	248	137	1.0
5	Female	20	4.83	114	104	1.5
6	Male	17	6.68	151	129	1.6

Note：Age（year）; WBC-White blood count($\times 10^9$/L)；Hb-Hemoglobin (g/L)；PLT-Platelet count ($\times 10^9$/L)；M-Monoblast, P-Promonocyte.CV:Control volunteers; IP: Incipient patient; NRP: Non remission patients; RP: Remission patients.
